# On Regeneration of Bone in the Human Subject after Excision

**Published:** 1844-10

**Authors:** 


Art. IV.—*Ueber Wiedererzeugung der Knochen nach Resectionen beim Menschen.* Von D. Kajetan Textor.—*Würzburg*, 1844.

*On Regeneration of Bone in the Human Subject after Excision.* By D. Kajetan Textor.—*Würzburg*, 1844. 8vo, pp. 26.

This little *brochure* constitutes a practical exposition of the advantages to be derived from partial excision of bone in many cases both of disease and traumatic injury, preferably to amputation or exarticulation. The author discusses the objections usually deterring surgeons from this operation, regarding them as untenable for the most part, or at least only as what will apply to any important procedure in surgery. In a very brief compass, we have here the value of the operation abundantly illustrated; and the successful results of the cases recorded in the present publication, amply bear out the author's commendations of the practice. The examples given of the reproductive power in bone after exsection, in many instances, are both curious and interesting, as well as important in the practical conclusions to which they lead. Altogether, we have to express our decided approval of this little pamphlet, and to assure its author that the profession will not fail to appreciate the importance of his contribution. We have him particularly to thank for letting us have the *matter* in a few words, and not, as is too much the fashion of the present day, obscuring valuable facts by superfluous garniture in order to make a *book*.

